# Gait Monitoring and Analysis: A Mathematical Approach

**DOI:** 10.3390/s23187743

**Published:** 2023-09-07

**Authors:** Massimo Canonico, Francesco Desimoni, Alberto Ferrero, Pietro Antonio Grassi, Christopher Irwin, Daiana Campani, Alberto Dal Molin, Massimiliano Panella, Luca Magistrelli

**Affiliations:** 1 Department of Sciences and Technological Innovation, University of Piemonte Orientale, 15121 Alessandria, Italy; francesco.desimoni@uniupo.it (F.D.); alberto.ferrero@uniupo.it (A.F.); pietro.grassi@uniupo.it (P.A.G.); christopher.irwin@uniupo.it (C.I.); 2 Department of Translational Medicine, Università del Piemonte Orientale, 28100 Novara, Italy; daiana.campani@uniupo.it (D.C.); alberto.dalmolin@uniupo.it (A.D.M.); massimiliano.panella@uniupo.it (M.P.); luca.magistrelli@maggioreosp.novara.it (L.M.)

**Keywords:** cloud computing, wearable devices, gait monitoring, telemedicine

## Abstract

Gait abnormalities are common in the elderly and individuals diagnosed with Parkinson’s, often leading to reduced mobility and increased fall risk. Monitoring and assessing gait patterns in these populations play a crucial role in understanding disease progression, early detection of motor impairments, and developing personalized rehabilitation strategies. In particular, by identifying gait irregularities at an early stage, healthcare professionals can implement timely interventions and personalized therapeutic approaches, potentially delaying the onset of severe motor symptoms and improving overall patient outcomes. In this paper, we studied older adults affected by chronic diseases and/or Parkinson’s disease by monitoring their gait due to wearable devices that can accurately detect a person’s movements. In our study, about 50 people were involved in the trial (20 with Parkinson’s disease and 30 people with chronic diseases) who have worn our device for at least 6 months. During the experimentation, each device collected 25 samples from the accelerometer sensor for each second. By analyzing those data, we propose a metric for the “gait quality” based on the measure of entropy obtained by applying the Fourier transform.

## 1. Introduction

The world’s population is rapidly aging, and the number of older adults will increase significantly in the coming decades [1]. As individuals age, maintaining physical function and independence becomes crucial to their overall well-being. One key indicator of an individual’s physical function is their gait, which refers to the manner of walking or the pattern of movement during locomotion. Monitoring gait in older people holds significant importance as it can provide valuable insights into their overall health, risk of falls, and potential mobility limitations. Moreover, gait analysis serves as a noninvasive and cost-effective method to assess various age-related conditions and diseases affecting the musculoskeletal and neurological systems.

Gait analysis has long been recognized as a valuable tool in clinical research and rehabilitation settings [2]. By objectively evaluating gait parameters, such as stride length, gait speed, and cadence, healthcare professionals can gain critical insights into an individual’s mobility and balance. Monitoring gait patterns over time allows for detecting subtle changes that may signify early signs of decline or underlying health conditions. Such changes can range from minor alterations in walking speed to more pronounced abnormalities in gait rhythm or symmetry.

Falls represent a significant concern for older adults, as they can lead to severe injuries and loss of independence. Research indicates that gait abnormalities are often associated with an increased risk of falls among the elderly population [3,4]. By closely monitoring gait, healthcare providers can identify those at higher risk and implement appropriate interventions to prevent falls. Moreover, gait analysis can help identify specific gait parameters that may contribute to fall risk, enabling targeted interventions tailored to individual needs [5,6].

Furthermore, gait analysis provides insights into the functional decline associated with age-related musculoskeletal and neurological disorders. Conditions such as osteoarthritis, Parkinson’s disease, stroke, and peripheral neuropathy can significantly impact an individual’s gait pattern [7]. Continuous monitoring of gait parameters in these populations allows for the early detection of functional decline and facilitates the development of targeted interventions to mitigate the negative effects on mobility and overall quality of life.

In addition to diagnosing and managing specific conditions, monitoring gait in the elderly population can serve as a proactive approach to maintaining overall health and independence. By establishing baseline gait parameters for individuals, healthcare providers can track changes over time, enabling early interventions and personalized treatment plans.

Summing up, monitoring gait in the aging population plays a crucial role in promoting health, independence, and well-being. By evaluating gait parameters, healthcare professionals can gain valuable insights into an individual’s overall health status, detect early signs of decline, identify fall risks, and manage age-related conditions affecting mobility. Emphasizing the importance of gait analysis in clinical practice and research will enable the development of targeted interventions aimed at improving the functional mobility and independence of older adults. In this paper, we show the partial results we obtained by monitoring the gait of 20 people affected by Parkinson’s disease. We also define a new value related to the “quality of gait” by exploiting the Fourier analysis applied to the data obtained in the experimentation. The purpose of this paper is to provide methodological insight related to the mathematical model adopted for the analysis of the data obtained. The TED study is still in progress [7]. The final results of the study will be published in a subsequent paper.

The paper is organized as follows: In Section 2, we discuss the related works proposed in the scientific literature, while in Section 3, we illustrate the system architecture. In Section 4, we propose the data analysis used to quantify the gait quality; in Section 5, we discuss the mathematical approach and the gait description from a physical point of view. In Section 6, we discuss the partial results obtained. Finally, in Section 7, we conclude the paper and we outline our future works.

## 2. Related Works

In the last few years, there have been several studies aimed at analyzing the gait of people, in particular of the fragile ones. To the best of our knowledge, most of them exploit wearable, although cumbersome, devices that are only suitable for short sessions in a lab environment but are uncomfortable to wear for a long time and unsuitable to be used in free-living conditions.

In particular, a study proposed by the Politecnico di Torino [8] employs shoes with sensorized insoles and a chest-mounted device, both used to record data in a controlled laboratory setting. Other studies adopt video-based solutions [9] or different kinds of external systems, such as sensorized walkways [10]. Unfortunately, forcing a person to wear different devices or interact with unfamiliar technology to obtain accurate data from their gait could compromise the data themselves since the person can be uncomfortable and change the way he or she moves. Moreover, when a device makes the user uncomfortable, it can only be used for short continuous sessions, usually a few hours at best, thus entailing two main problems: to gather statistically meaningful amounts of data, several sessions need to take place, and short sessions are unlikely to feature relevant events such as stumbles and falls. The intensity of the symptoms caused by the disorders affecting the patients may vary during a given day, week, or year. Short and infrequent sessions could miss important fluctuations or key events and not fully represent the patient’s real condition.

Smaller and lighter devices can also create issues if the patient is not used to them and can only be used in a specific laboratory environment under qualified personnel supervision. In [11], the patients wore an inertial sensor attached to a waist-mounted strap situated on the lower back while performing a supervised timed up and go test to extract useful features from the signal for fall risk assessment. In [12], the authors placed a small accelerometer on the patients’ lower back before asking them to perform a series of tasks in a supervised laboratory environment to identify early markers of Parkinson’s disease (PD). Laboratory environments usually have smooth and flat surfaces without external influences, making these conditions significantly different from the ones people find in their daily lives, characterized by irregular surfaces and obstacles. The presence of other people can also make the patients feel anxious.

The data collected in these circumstances, for the reasons presented above, may not represent the real condition of the patients, who can be prone to alter their actions subconsciously and, thus, their gait. Many studies attempted to use more widespread and accessible devices, such as torso-mounted smartphones [13,14,15], which can accurately capture the forces involved in a walk with minimal noise from other sources. However, such devices still encounter low acceptance on behalf of the patients due to their weight, size, location, or stigmatization [16,17]. Smartwatches, like other wrist-worn devices and accessories, are significantly easier to wear, less bulky, and more likely to be accepted by the user. Unsurprisingly, the scientific literature has been moving in this direction for a few years. The goals in this scope are many and diverse.

In [18], the authors exploited the acceleration data available in the UK Biobank database to determine whether acceleration data collected during daily living can serve as a prodromal marker for PD. Although the number of subjects is extremely high, the authors only focused on generic information extracted from the data, such as average acceleration, without analyzing the patients’ gait. Another study using data from the same database is [19], in which the goal was to use free-living acceleration data to detect PD early. The authors split the raw data into segments of either gait or low acceleration magnitude and found notable differences between PD patients and healthy controls. Both studies only focused on PD without proposing a way to track a patient’s health status applicable to a more general population. Other studies, such as [20], also analyzed the gait for an entirely different reason. The authors wanted to extrapolate gait features from the acceleration data, which could authenticate the user wearing the device. While this study represents additional proof of how gait features can be observed at the wrist level, the data were collected by having subjects walk a corridor six times at three different paces, thus creating a controlled environment. Moreover, the study did not focus on the health status of the subjects or the quality of their gait.

A key aspect of gait analysis is differentiating what the user is doing, a task known as activity recognition. Studies such as [21,22] are useful in this scope. In the first one, the authors aimed at detecting gait segments in acceleration data collected in real-life scenarios. In contrast, the second one evaluates activity recognition and fall detection methods on different datasets. While understanding what the user is doing and isolating gait sections is the first step of assessing gait quality, the studies did not explore which features could be extracted from these sections and actions to obtain an approximate measure of the quality of the movements or the user’s health status.

In [23], the authors investigated the correlation between wrist movements and freezing events in patients with PD. This event detection is more closely related to health status than the two previous studies. However, the proposal is still too specific to be applied to a larger population. To create a reliable process to extrapolate features and detect gait events from acceleration data, the authors of [24] proposed a deep-learning-based automatic pipeline validated against a sensorized walkway. Although studies such as this one offer an interesting foundation, they do not explore how the features can be used to assess quality and health status.

One of the primary purposes of our article is precisely to identify some indices that can give some information on the gait quality and, hence, on the progress of the disease. Helpful information can be obtained by performing a spectral analysis of the data coming from body sensors. This is performed by applying the Fourier transform to the acceleration data. This procedure allows us to compute some indicators, such as the mean value of the frequency, its variance, and its entropy. Having the data of single individuals over a pretty long period of time, the variation of those indicators can give information on the progress of the disease. We now quote some recent articles in which a Fourier analysis is performed on data coming from human body sensors.

In [25], the authors performed a Fourier analysis of the stride-by-stride estimates of the linear acceleration coming from body-worn inertial sensors. The forces measured by the triaxial accelerometer were transposed from a local to a global frame using a quaternion-based orientation estimation algorithm and by detecting when each stride began using a gait segmentation algorithm. The results contained in [25] could be useful in future developments of our research about analysis of data coming from subjects with neuromuscular disorders, as we explain in more detail in Section 5 about the physical description of our analysis. The authors of [26] developed models for reducing the complexities of extracting features from data coming, among others, from wearable sensors. The extraction of the features is based on spectral analysis, and, in turn, the frequency analysis is based on the fast Fourier transform. Additionally, in [27], a method for analyzing data from a human body sensor was performed. It developed an algorithm that could identify the gait behavior with high precision. As in the previous ones, also in [27], the Fourier transform represents a fundamental tool in the classification process. Other applications of the Fourier transform for the analysis of frequencies can be found in [28], where the authors studied the data coming from people having different kinds of neuromuscular disorders. The authors of [28] developed software that could detect quantitative observations that could improve the subjects’ gait. For other details about the applications of the Fourier transform to human gait activity, see also the references contained in [25,26,27,28]. The relevance of our results compared with the existing ones is given by identifying some indicators, primarily the entropy, which constitutes a straightforward way to quantify the deterioration of an individual’s gait from specific neuromuscular diseases.

Reviewing the existing literature shows how the benefits of a wrist-worn sensor, such as better wearability, usually outweigh the drawbacks, such as the higher amount of noise in the data compared with other types of devices. With its growing capabilities, machine learning can also help mitigate the effect of the noise when used in data processing and analysis tasks. This way, patients are more comfortable, and collecting data in real-life scenarios becomes easier overall. Although multiple studies use wrist-worn sensors, a wrist-based gait quality assessment does not seem to be a recurring topic. One of the studies that attempted to fill this gap is [29], in which the authors focused on daily-living gait quality and quantity in patients with Huntington’s disease (HD). Since HD leads to altered gait patterns, the same methods could be used to detect a decrease in gait quality in a larger population. Despite this proposal, the existing literature still lacks an index that can be extrapolated from the acceleration signal and applied to a diverse population of patients, representing a variation in gait quality over time. We propose a method that, albeit similar to the last analyzed study, constitutes an alternative process and a different measure of gait quality to improve the assessment of a variation in health status.

## 3. System Architecture

**Figure 1:**
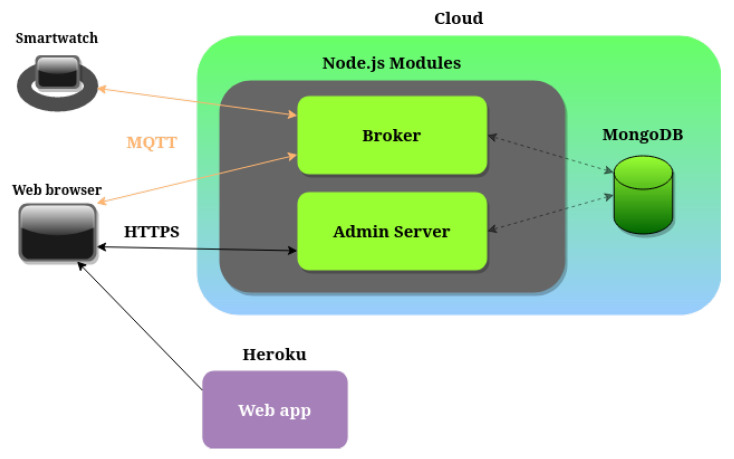
System architecture. The figure shows the smartwatch used for data collection, a web browser to access the web app on the left, and the two communication protocols used. On the right side, the figure shows the server side of the system consisting of two Node.js modules, a MongoDB database, and the Heroku platform used to host the web application.

The data used for the analysis were gathered during two similar studies [7], which required two slightly different, albeit equivalent, architectures. The shared components and the ones on which they differ are shown in Figure 1 and detailed below.

**Figure 2:**
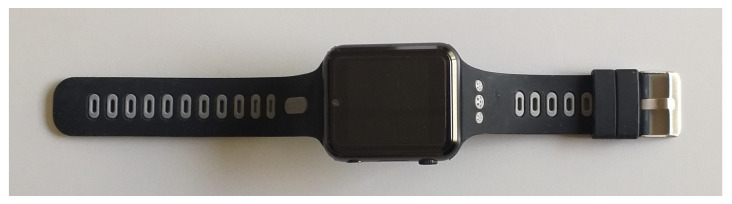
Android smartwatch used for data collection.

Two distinct types of wearable devices were employed for data collection, but since the collected data are comparable, only one is described for brevity. The device is an Android smartwatch (see Figure 2) with an embedded accelerometer that can send samples to the server using the built-in GSM module without connecting to a nearby device. A custom app was developed and installed on the smartwatches to collect and send the data. The main server is built using Node.js modules and runs on an Ubuntu virtual machine hosted on Chameleon Cloud [30]. The modules provide the APIs and the means of communication to upload and retrieve the data sent through HTTP/HTTPS [31,32] or MQTT [33]. Once the data reach the server, MongoDB [34] is used to store them, chosen for its ability to store time series efficiently. The data stored on the server can be accessed and queried using a simple web application developed to ease the retrieval of the samples for research purposes and to monitor the status of the devices in the system. This dashboard application has been deployed to Heroku [35] to reduce hosting-related work.

## 4. Data Analysis

This section will describe aspects of analyzing the data collected during the experiments. In our experimentation, about 50 people were involved (20 people with Parkinson’s disease and 30 people with chronic diseases) who have worn our device for at least 6 months each day. The usage of the device for every single person changed based on the tolerability of the device itself—the device collected at least 25 sample values from the accelerometer sensor each second. The total amount of data collected are about 40 GBytes.

The study’s main objective is to assess the gait quality over time using the sensor data. To achieve this, we developed a mathematical method based on Fourier analysis [36] that can give a relative measure of the quality of the walk. This spectral analysis of the data has revealed itself to be an efficient tool for detecting the variation of the amplitude of the signals corresponding to the different frequencies. This kind of frequency variation over time can be interpreted as a symptom that something is changing in the gait quality. This suggests a possible strategy for detecting suitable indices that could detect a possible deterioration in gait quality; see Section 5 for details.

However, since our users did not have any indication of when to wear the device, it is also important to find a way to classify the user’s activity based on the data coming from the sensors. To this end, we built a machine learning model for human activity recognition (HAR) classification. The following sections will describe the mathematical method for obtaining gait quality scores and the machine learning model for HAR classification.

### 4.1. Gait Analysis

**Figure 3:**
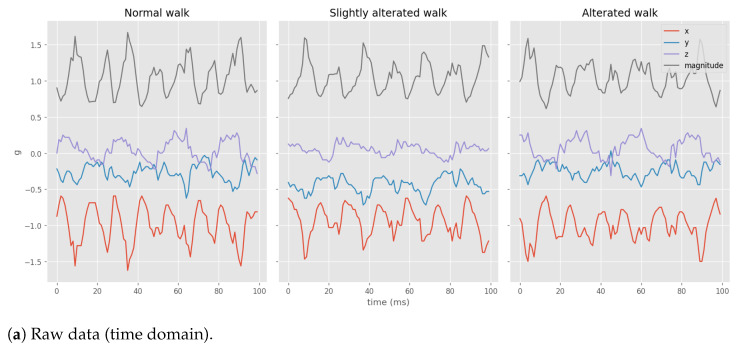

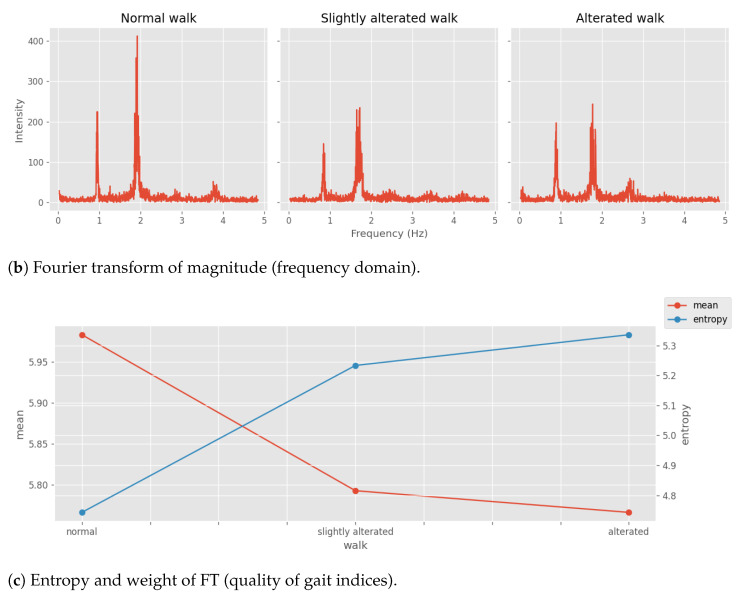
Data of the controlled walks. Each figure showcases plots corresponding to different levels of degradation. (**a**) illustrates the acceleration intensity along the xyz-axis and the magnitude in the time domain. (**b**) presents a selected segment of the Fourier transform for the three walks. Lastly, (**c**) reveals the trends of entropy and the weighted mean of the Fourier transform, which were used as gait indices.

This section will analyze the approach used to determine gait quality through statistical indices. During the discussion, a reference will be made to data collected in a controlled environment using the same methods and sensor employed during the user experimentation (Figure 3). Specifically, three controlled walks were performed, including a *baseline* walk and two walks with varying levels of alteration.

The goal is to find indices of gait quality using only the data from the accelerometer worn by the participants in the experiment. As previously mentioned, the sensor used in the experiments provides measurements of acceleration intensity along the XYZ-axes (with a scale range of ±4 g) and the associated timestamps (with a precision of 1 microsecond). For the analysis, we used the acceleration magnitude calculated as $mag=\sqrt{x^{2}+y^{2}+z^{2}}$, which provides a direction-independent measure description of acceleration intensity. Figure 3a shows an excerpt of the raw data from the controlled walks.

We employed an approach based on spectral analysis of the acceleration data to derive indices that quantify the gait quality over time. A qualitative measure of gait quality can be obtained by analyzing the spectrum obtained through the Fourier transform of the acceleration magnitude. The differences between walks are directly observable in Figure 3b, which compares the spectra of the *controlled walks*. In particular, it is possible to observe that the spectrum of the *baseline* walk (left) is characterized by a well-defined dominant frequency. In contrast, this frequency degrades in the altered walks (center and right), accompanied by the appearance of other minor frequencies, resulting in a noisier spectrum.

Based on these observations, it was possible to introduce indices capable of quantifying the degree of gait alteration. Two main indices qualitatively describe gait: the weighted mean and the entropy of the Fourier frequencies (Figure 3c). The former tends to decrease when the walks degrade; the latter increases with walk degradation. These indices’ formal mathematical and physical descriptions are presented in Section 5.

### 4.2. HAR Classification

The classification model was constructed using a supervised learning approach employing an XGBoost classifier [37] model. The model was trained following a similar approach as described by Walmsley et al. in [38]. The accelerometer data of acceleration intensity along the 3-axes (x,y,z) with associated timestamps were segmented into non-overlapping 30-s windows. Feature extraction was then performed on each window, comprising the calculation of 38 distinct statistical indices characterizing the temporal window (Table 1 summarizes the features used and their type). Each window was annotated with the corresponding activity label.

However, due to constraints in our experimentation, obtaining labeled data from our sensors was not feasible on a scale sufficient to construct an appropriate dataset. Consequently, we adopted the publicly available *Capture24* dataset [39]. The model trained on this dataset was subsequently applied to our sensor data to accomplish the classification task. Several necessary modifications were made to align the model with our specific data and context. We subsampled the time series, transitioning from a sampling frequency of 100 to 25 Hz. Additionally, we retained only the samples related to the *walking*, *sleeping*, and *sit–stand* classes.

The resulting dataset was utilized for training the XGBoost classifier. During training, an optimal hyperparameter search was conducted using a cross-validation setting and stratified K-fold technique (to address the class imbalance within the dataset). This yielded a weighted F1-score of 87%.

## 5. Fourier Analysis of the Acceleration Data

The acceleration data collected by a device after a walk of duration *T* may be interpreted as a function of the time t∈[0,T]. We will perform a Fourier analysis of these data. Given a generic function *F*, we denote by $\hat{F}$ its Fourier transform:

$$\hat{F}\left(s\right)=\int_{-\infty }^{+\infty }e^{-2\pi ist}\;F\left(t\right)\;dt$$

where s∈(−∞,+∞) is the variable denoting frequencies. For more details on the Fourier transform and its applications, see [36]. Since the data coming from the device are discrete, we will use the fast Fourier transform as an approximation for the classical Fourier transform: given a sequence of *N* data $d_{1},\ldots ,d_{N}$, its fast Fourier transform is given by the sequence ${\hat{d}}_{1},\ldots {\hat{d}}_{N}$ defined by

$${\hat{d}}_{k+1}=\sum_{j=0}^{N-1}e^{-\frac{2\pi i}{N}\;jk}\;d_{j+1}\;\mathrm{for}\;\mathrm{any}\;k=0,\ldots ,N-1\;.$$

Suppose that the device collects *N* data in the period of time *T* at time steps of constant length δ. If the function *F* represents the acceleration data, then putting $d_{j+1}=F\left(j\delta \right)$ for j=0,…,N−1 and $s_{k}=k/T$ for k=0,…,N−1, then

(1) $$\begin{array}{cc} \hat{F}\left(s_{k}\right) & \approx \delta {\hat{d}}_{k+1}\;\mathrm{for}\;\mathrm{any}\;k=0,\ldots ,N-1\;. \end{array}$$

Formula (1) can be considered meaningful only for small values of $s_{k}$ since the approximation of the integral in the second line of (1) can be regarded as accurate only when the coefficient $\frac{2\pi k}{N\delta }$, appearing in the complex exponential, is not too large. Roughly speaking, it is reasonable to consider values of *k* not exceeding $\frac{N}{2}$. We now utilize the above mathematical description to outline some of the indicators of the quality of walking performances. A first possibility could be to evaluate the quantity *E* defined as the mean value of the kinetic energy along the whole walk. Letting $\vec{a}\left(t\right)=\left(a_{x}\left(t\right),a_{y}\left(t\right),a_{z}\left(t\right)\right)$ the vector acceleration and *m* the person’s mass, we have that

$$\begin{array}{cc} E\; & =\frac{m\left(|{\hat{a}}_{x}{\left(0\right)|}^{2}+|{\hat{a}}_{y}{\left(0\right)|}^{2}+{\left|{\hat{a}}_{z}\left(0\right)\right|}^{2}\right)}{8}\\ \; & \;+\frac{m}{8\pi^{2}T^{2}}\;\sum_{k\in Z\setminus \{0\}}\;\left(\frac{|{\hat{a}}_{x}\left(0\right)-{\hat{a}}_{x}\left(s_{k}\right){|}^{2}}{s_{k}^{2}}+\frac{+|{\hat{a}}_{y}\left(0\right)-{\hat{a}}_{y}\left(s_{k}\right){|}^{2}+{\left|{\hat{a}}_{z}\left(0\right)-{\hat{a}}_{z}\left(s_{k}\right)\right|}^{2}}{s_{k}^{2}}\right)\;. \end{array}$$

Unfortunately, the mean values of the kinetic energy along the entire walk, obtained from the data of the device, are heavily influenced by the presence of components of gravity, thus producing very large values (of the order of $10^{9}\;J$), which have nothing to do with the real values coming from a walking person. The main difficulty in removing the contribution of gravity is that the device measures its components in the frame of reference of the device, which is moving together with the part of the body where it is mounted. A possible solution to this problem is considering other devices containing a gyroscope. At the present state of the art, we do not have such kind of data since the data collected in past months come from a simple accelerometer. Among our purposes for future works is to manage data from a device containing a gyroscope. Energy could be a significant performance indicator. The results contained in [25] could be very useful in managing data from an accelerometer incorporating the contribution of gravity.

In the present work, we focus our attention essentially on the frequencies that characterize a generic walk as a performance indicator of the quality of walking. More precisely, we notice that a generic walk is characterized by a specific frequency $s_{k}$ and that, in general, it is not a single frequency but rather a small open set of frequencies centered around $s_{k}$. In our data analysis, we choose as a function *f* the modulus of the acceleration measured by the device. The corresponding coefficient $\hat{f}\left(s_{k}\right)$ is the most relevant among the other frequencies $|\hat{f}\left(s_{k}\right)\left|>>\right|\hat{f}\left(s_{k}^{\prime }\right)|$ if $k^{\prime }\neq k$. This emerges from the data analysis: there is a small collection of frequencies around a peak that are relevant for a given walk, and we observe that the decreasing quality of the walk is characterized by the appearance of new lower frequencies in the spectrum.

We recall here the main idea before dwelling on mathematics. The FT of the gait leads to a noisy spectrum characterized by features of the walking gestures. Nonetheless, there is a dominant frequency, which is the fundamental cyclic characteristic of the gait. Then, from the experimental observation of the data, we noticed that the intensity of the fundamental frequency lowered, and new relevant lower modes were emerging on the altered walks. The combined effects are interpreted as the main difference between the noisy spectra of normal and altered ones. With this observation, we verify in mathematical formulas whether the two indicators (the variation of the mean and the variation of the entropy) describe the decreasing quality of the walks.

We analyze the system as follows: we vary the coefficients $\hat{f}\left(s_{k}|\beta \right)$ concerning variable β, which denotes the progression of the disease or temporally registers the different walks; then, we introduce the following quantity:

$$P\left(\beta \right)=\sum_{k=0}^{+\infty }{\left|\hat{f}\left(s_{k}|\beta \right)\right|}^{2}$$

and we assume that it remains constant as the parameter β varies.

Given a generic sequence of data ${\left\{y_{k}\right\}}_{k\geq 0}$, we introduce the following weighted mean value defined by

$$\langle y_{k}\rangle :=\sum_{k=0}^{+\infty }y_{k}\;{\left|\hat{f}\left(s_{k}|\beta \right)\right|}^{2}\;.$$

The dependence upon $s_{k}$ is only through $\hat{f}\left(s_{k}|\beta \right)$. It has been observed (see Section 4) that the variation of the height of the peak of the principal frequency, with a coefficient $\hat{f}\left(s_{k}|\beta \right)$, characterizes the quality of the walk together with the appearance of a new relevant frequency $k^{\prime }$, with a coefficient $\hat{f}\left(s_{k^{\prime }}|\beta \right)$, such that

$$|\hat{f}\left(s_{k}|\beta \right)\left|\;\geq \;\right|\hat{f}\left(s_{k^{\prime }}|\beta \right)\left|\;>>\;\right|\hat{f}\left(s_{k^{\prime \prime }}|\beta \right)|\;,$$

where $s_{k^{\prime \prime }}$ are the irrelevant frequencies, namely, those highly suppressed in the spectrum of the walks.

To analyze the quality of the walk, we study the variation of the mean value of the frequencies and of the variance defined in the following way:

(2) $$\begin{array}{ccc} \;\mu_{s}\left(\beta \right) & := & \langle s_{k}\rangle =\sum_{k=0}^{+\infty }s_{k}{\left|\hat{f}\left(s_{k}|\beta \right)\right|}^{2}\;,\;\\ \sigma_{s}^{2}\left(\beta \right) & := & \langle s_{k}^{2}\rangle -\mu_{s}^{2}\left(\beta \right)=\sum_{k=0}^{+\infty }s_{k}^{2}{\left|\hat{f}\left(s_{k}|\beta \right)\right|}^{2}-\mu_{s}^{2}\left(\beta \right)\;, \end{array}$$

in the different series of walk data.

If we compute the variation of P(β) and $\mu_{s}\left(\beta \right)$ with respect to β, we obtain

(3) $$\begin{array}{ccc} \partial_{\beta }P\left(\beta \right) & = & \sum_{k=0}^{+\infty }\partial_{\beta }{\left|\hat{f}\left(s_{k}|\beta \right)\right|}^{2}\;,\\ \;\partial_{\beta }\mu_{s}\left(\beta \right) & = & \sum_{k=0}^{+\infty }s_{k}\partial_{\beta }{\left|\hat{f}\left(s_{k}|\beta \right)\right|}^{2}\;. \end{array}$$

Setting $\partial_{\beta }P\left(\beta \right)=0$, i.e., P=P(β) does not depend on β, which is based on the assumption that there is no change in this quantity of the candidate during the entire data series; we assume that there is a new (the analysis can be performed assuming several new frequencies are appearing, but the model can be easily discussed in terms of a single new frequency) frequency $s_{k^{\prime }}$ appearing in the analysis of the series of walks, namely, changing β. This means that $\partial_{\beta }{\left|\hat{f}\left(s_{k^{\prime }}|\beta \right)\right|}^{2}>0$ or equivalently that $|\hat{f}\left(s_{k^{\prime }}|\beta \right){|}^{2}$ increases as β increases, but this implies also

(4) $$\begin{array}{c} \sum_{k\geq 0,\;k\neq k^{\prime }}\partial_{\beta }|\hat{f}\left(s_{k}|\beta \right){|}^{2}=-\partial_{\beta }{\left|\hat{f}\left(s_{k^{\prime }}|\beta \right)\right|}^{2}<0 \end{array}$$

and therefore the sum of the variations of $|\hat{f}\left(s_{k}|\beta \right){|}^{2}$ when $k\neq k^{\prime }$ decreases. This is seen in the data series during several walks, where we observe a progressive decrease in the quality of the walking act, resulting in low-frequency modes absent at the beginning of the experiment.

With a new frequency $s_{k^{\prime }}$, thanks to (3) and (4), we can write the variation of the mean as follows:

(5) $$\begin{array}{c} \partial_{\beta }\mu_{s}\left(\beta \right)=\sum_{k\geq 0,\;k\neq k^{\prime }}\left(s_{k}-s_{k^{\prime }}\right)\partial_{\beta }{\left|f\left(s_{k}|\beta \right)\right|}^{2}\;, \end{array}$$

which implies that $\partial_{\beta }\mu_{s}\left(\beta \right)\leq 0$, i.e., a decreasing mean value if either $(s_{k}-s_{k^{\prime }})<0$ with $\partial_{\beta }{\left|f\left(s_{k}|\beta \right)\right|}^{2}>0$ or $(s_{k}-s_{k^{\prime }})>0$ with $\partial_{\beta }{\left|f\left(s_{k}|\beta \right)\right|}^{2}<0$ (here, we made a simplifying assumption; namely, we assume that all amplitudes $\partial_{\beta }{\left|f\left(s_{k}|\beta \right)\right|}^{2}$ decrease. Of course, that might not be the case, but since we assume that there is a narrow set of frequencies around the main frequency of the walk, we can assume that they are all decreasing). Notice that if the amplitudes associated with the original (before the appearance of the new frequency $s_{k}$) frequencies are decreasing $\partial_{\beta }{\left|f\left(s_{k}|\beta \right)\right|}^{2}<0$, the mean value $\mu_{s}\left(\beta \right)$ decreases when $s_{k}<s_{k^{\prime }}$. Vice versa, if the amplitude associated with the original frequencies is increasing $\partial_{\beta }{\left|f\left(s_{k}|\beta \right)\right|}^{2}>0$, the mean value decreases if a new high frequency $s_{k}>s_{k^{\prime }}$ emerges. From the data, we see the first situation, namely, an overall decrease of the amplitudes $|f\left(s_{k}|\beta \right){|}^{2}$ and the appearance of a low frequency $s_{k^{\prime }}$. If we relax the condition about the power, $\partial_{\beta }P\left(\beta \right)=0$ and we assume $\partial_{\beta }P\left(\beta \right)\leq 0$, then we have a weaker condition instead of (5)

(6) $$\begin{array}{c} \partial_{\beta }\mu_{s}\left(\beta \right)\leq \sum_{k\geq 0,\;k\neq k^{\prime }}\left(s_{k}-s_{k^{\prime }}\right)\partial_{\beta }{\left|f\left(s_{k}|\beta \right)\right|}^{2}\;, \end{array}$$

but we can conclude the same features as above.

For what concerns the variance $\sigma_{s}^{2}$, we compute the variation with respect to β and we obtain

(7) $$\begin{array}{c} \partial_{\beta }\sigma_{s}^{2}\left(\beta \right)=2\sum_{k\geq 0,\;k\neq k^{\prime }}\left(s_{k}-s_{k^{\prime }}\right)\left(\frac{s_{k}+s_{k^{\prime }}}{2}-\mu_{s}\left(\beta \right)\right)\partial_{\beta }{\left|f\left(s_{k}|\beta \right)\right|}^{2} \end{array}$$

which shows that it has a similar behavior of $\partial_{\beta }\mu_{s}\left(\beta \right)$, because of the product $\left(s_{k}-s_{k^{\prime }}\right)\partial_{\beta }{\left|f\left(s_{k}|\beta \right)\right|}^{2}$ appearing as in $\partial_{\beta }\mu_{s}\left(\beta \right)$, but the factor $\left(\frac{s_{k}+s_{k^{\prime }}}{2}-\mu_{s}\left(\beta \right)\right)$ makes the analysis rather critical since the mean value $\mu_{s}\left(\beta \right)$ could be either greater or lower than the arithmetic mean of $s_{k}$ and $s_{k^{\prime }}$. However, it is reasonable to think that $\left(\frac{s_{k}+s_{k^{\prime }}}{2}-\mu_{s}\left(\beta \right)\right)$ is rather close to zero, and therefore, the variation of the variance is rather mild. Indeed, from the data, the variance variation is relatively mild because of that factor.

We can study a third quantity, namely, the entropy *S*. This is defined as follows: since $|f\left(s_{k}|\beta \right){|}^{2}$ are positive, we can define the amplitudes

$$\rho \left(k|\beta \right)=\frac{|\hat{f}\left(s_{k}|\beta \right){|}^{2}}{P(\beta )}$$

such that $\sum_{k=0}^{+\infty }\rho \left(k|\beta \right)=1$; then we set

(8) $$\begin{array}{c} S=-\sum_{k=0}^{+\infty }\rho \left(k|\beta \right)\ln\rho \left(k|\beta \right)\;. \end{array}$$

Assuming that $\partial_{\beta }P\left(\beta \right)=0$ and exploiting (4), for the variation of the entropy under β, we obtain the following equation:

(9) $$\begin{array}{c} \partial_{\beta }S=-\frac{1}{P(\beta )}\;\sum_{k\geq 0,\;k\neq k^{\prime }}\ln\left[\frac{\rho (k|\beta )}{\rho (k^{\prime }|\beta )}\right]\partial_{\beta }{\left|\hat{f}\left(s_{k}|\beta \right)\right|}^{2}\;. \end{array}$$

As we have seen above, if $\partial_{\beta }{\left|\hat{f}\left(s_{k}|\beta \right)\right|}^{2}\leq 0$, which means that the amplitudes of the original frequencies are decreasing, and if we assume that $\rho \left(k|\beta \right)>\rho \left(k^{\prime }|\beta \right)$, which means that the amplitude $\rho (k^{\prime }|\beta )$ associated with the new frequency is lower than the original one, then the entropy increases $\partial_{\beta }S\geq 0$. This is consistent with the fact that the overall quality of the walk is getting lower with the spreading of the original narrow-peaked spectrum around the main frequency of the walk.

## 6. Experimental Results

The results obtained by the two studies are presented below. Note that the change in gait quality throughout the study was synthetically represented as the percentage variation of entropy between the initial and final periods.

**Figure 4:**
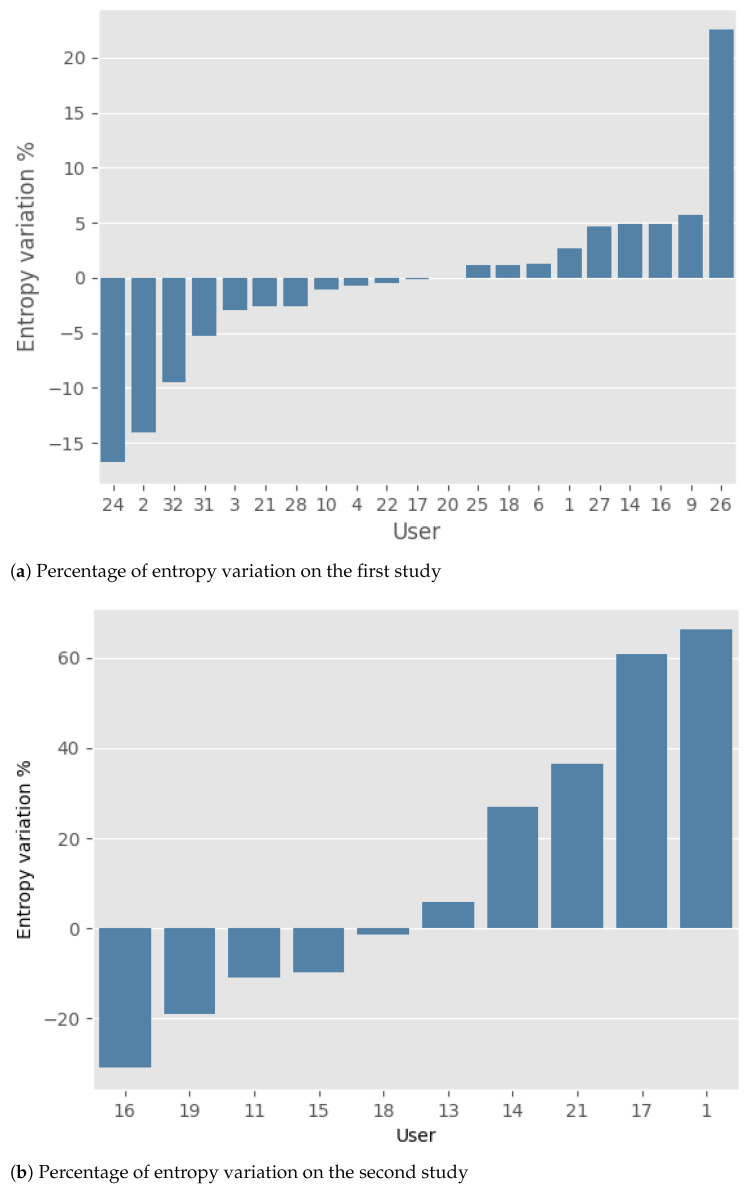
Results of the two studies. Every study’s percentage of entropy variation (*y*-axis) between the first and final week of data collection is reported per user (*x*-axis). In both studies, we applied a selection criterion to retain only those participants who conducted a minimum of 10 walks during the data collection period.

Regarding the first study (Figure 4a), it is possible to observe that, in general, the number of improvements (11 participants) and deteriorations (10 participants) are basically the same. Moreover, the mean percentage of improvement and deterioration is nearly identical: 5.1% (±5.8) and 4.9% (±6.5), respectively. For a comprehensive overview of the results, please refer to Table 2.

In the second study (Figure 4b), an equal number of deteriorations and improvements can still be observed. However, the average magnitude of deterioration, 39.2% (±24.9), is higher than that of improvement, 14.5% (±11.2). To give at least a weak statistical significance to this fact, we introduce the two random variables *I* (improvements) and *D* (deteriorations) corresponding to the absolute value of the entropy variations (percentage) in the two groups, respectively. Performing a logarithmic transformation and applying an estimate for the difference of the means for unpaired data (see Chapters 12–13 in [40]), we obtain the following estimate for the ratio of the geometrical means $\mu_{D}$ and $\mu_{I}$:

$$1.15<\frac{\mu_{D}}{\mu_{I}}<7.84\;(C.I.\;\;\;80\;\%)\;.$$

The confidence level is unavoidably low due to the small number of data in the sample (only 10 users). Please refer to Table 3 for a comprehensive overview of the results.

**Figure 5:**
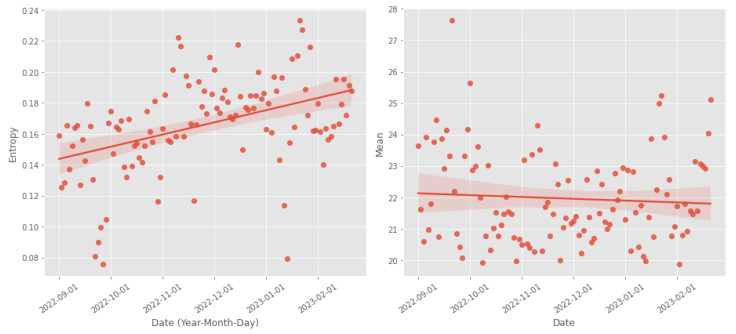
Gait index values and trends for participant 14. Each data point on the graph corresponds to an individual walk within the time frame indicated along the *x*-axis. The walks are spaced 1 day apart. Additionally, a regression line and the 95% confidence interval have been incorporated for both plots. The plot on the left illustrates the weighted mean, while the entropy value is displayed on the right.

We also conducted a more in-depth study on one of the participants. Participant 14 from the second study used the device once daily while walking. This allowed for more precise monitoring of gait progression without relying on the classifier. The results of this case study are shown in Figure 5. In this case, we can observe a deterioration in gait quality, evidenced by an increase in entropy and a decrease in mean. The observed results were partially confirmed through an interview with the participant. During the study, the participant willingly responded to specific questions regarding their perception of increased difficulty in walking and whether they noticed any changes in their gait. From the interview, it emerged that the participant declared a deterioration in the quality of walking, which is compatible with what emerges from the analysis of the experimental data.

## 7. Conclusions

In this paper, we monitor and analyze the gait of older adults affected by chronic diseases and/or Parkinson’s disease. Gait analysis is a valuable tool since it can detect the early signs of declining health conditions.

In particular, by exploiting a wearable device with an accelerometer sensor that sends data to our private cloud infrastructure, we collected 25 data samples for each second from 50 people for 6 months (more than 40 GBytes of data have been analyzed). By exploiting those data, we propose a metric for the “gait quality” based on the measure of entropy obtained by applying the Fourier transform.

By computing the percentage of entropy variation, we have noted that during the trial, some people had a decline of their gait quality (the entropy increased), other people had an improvement of their gait quality (the entropy decreased), and for some people, we have not seen any significant difference in their gait quality (the entropy is stable). The results obtained have been confirmed by the medical staff by visiting and interviewing the patients, thus validating our approach. We believe that this kind of approach, based on the research of suitable indicators of gait quality, could be very useful in determining for example, the progress of neuromuscular diseases. At the present state of the art, we identified three parameters of some interest that are able to determine a variation in the distribution of the fundamental frequencies over time: the mean value $\mu_{s}$, the variance $\sigma_{s}^{2}$, and the already-mentioned entropy *S*; see Section 5 for more details. In Section 5, we give a mathematical explanation of the reason for these indicators’ change when the quality of the gait declines over time. Indeed, we observed in the data of some individuals that the progress of their disease was accompanied by a variation in the distribution of the frequencies and, in particular, with a reduction of the amplitude of the main frequency and a simultaneous appearance of new peaks on lower frequencies. The decrement of the mean and the increment of the entropy with the progress of the disease find in Section 5 a rigorous mathematical explanation. The contents of the present article have to be considered only as initial results, and we believe that future studies will be necessary to find universal indicators of the quality gait; indeed, the three indicators considered in the present article only give information on the quality gait of a single person, and they have to be considered subjective of that particular individual and, hence, not comparable with the ones of other individuals.

As already explained in Section 2 in more detail, different from other works, the data collected during the preparation of this paper come from quite comfortable devices that can be worn by an individual for most part of a single day and for more consecutive days, even in the case of long periods of time (weeks or months). The positive aspect of this approach is that we can collect data from usual life, and this increases the probability of detecting special events such as a fall and analyzing the data just a few instants before that event. Moreover, the way the individual moves is not affected by wearing uncomfortable devices, and his or her behavior appears more natural than in a situation where he or she is forced to provide the data during a short session in a lab environment. The ones listed above are some of the reasons that encouraged us to choose this kind of approach in collecting and analyzing data.

However, in the future, we plan to compare our approach with similar studies. As mentioned in Section 2, to the best of our knowledge, other studies concerning gait monitoring and analysis exploit significantly different devices with respect to ours (such as shoes with sensorized insoles and a chest-mounted device), and consequently, the data provided by the sensors are hardly comparable with ours. To compare our solution with what was provided by other studies, we need to use our devices and the devices from other studies with the same people. For this reason, we are writing a project with researchers of Politecnico of Turin where we will compare our solutions with their solutions described in the paper [8]. In these new experiments, we plan to improve the accuracy of our solution by also taking advantage of a gyroscope sensor. Moreover, by exploiting machine learning algorithms, we plan to forecast the gait quality to prevent dangerous events, such as falls.

## Figures and Tables

**Table 1:** Features used to train the classification model.

Feature Type	Features
Position	xmin, xq25, xmed, xq75, xmax, ymin, yq25, ymed, yq75, ymax, zmin, zq25, zmed, zq75, zmax
Correlation	xycorr, yzcorr, zxcorr
Statistical	min, q25, med, q75, max
Motion	rollg, pitchg, yawg, avgroll, avgpitch, avgyaw, sdroll, sdpitch, sdyaw
Signal	pentropy, f1, f2, p1, p2, numPeaks, peakPromin

**Table 2:** Results of the first study. The table presents each participant’s minimum, maximum, and mean entropy values. It also includes the entropy values for the initial and final period of the study and the percentage variation of entropy.

Participant	Min Entropy	Max Entropy	Mean Entropy	Initial Entropy	Final Entropy	Entropy Variation %
1	0.682	0.755	0.718	0.707	0.726	2.694
2	0.672	0.842	0.766	0.782	0.672	−14.079
3	0.695	0.919	0.772	0.78	0.757	−2.921
4	0.706	0.825	0.756	0.755	0.75	−0.746
6	0.706	0.738	0.722	0.729	0.738	1.25
9	0.719	0.78	0.745	0.719	0.76	5.676
10	0.67	0.793	0.733	0.754	0.746	−1.068
14	0.665	0.755	0.713	0.691	0.725	4.841
16	0.688	0.798	0.75	0.72	0.754	4.846
17	0.659	0.718	0.683	0.684	0.683	−0.101
18	0.642	0.773	0.709	0.706	0.714	1.156
20	0.68	0.723	0.697	0.695	0.696	0.041
21	0.708	0.918	0.752	0.743	0.724	−2.576
22	0.676	0.814	0.715	0.73	0.727	−0.494
24	0.636	1.111	0.783	0.765	0.636	−16.771
25	0.638	0.748	0.711	0.703	0.711	1.138
26	0.674	0.86	0.712	0.702	0.86	22.495
27	0.637	0.781	0.713	0.723	0.757	4.658
28	0.718	0.812	0.756	0.773	0.753	−2.529
31	0.697	0.842	0.753	0.754	0.714	−5.306
32	0.682	0.923	0.742	0.788	0.714	−9.422

**Table 3:** Results of the second study. The table presents each participant’s minimum, maximum, and mean entropy values. It also includes the entropy values for the initial and final period of the study and the percentage variation of entropy.

Participant	Min Entropy	Max Entropy	Mean Entropy	Initial Entropy	Final Entropy	Entropy Variation %
1	0.081	0.281	0.185	0.143	0.238	66.238
11	0.044	0.184	0.109	0.137	0.122	−10.972
13	0.036	0.145	0.067	0.044	0.047	5.746
14	0.006	0.233	0.144	0.145	0.184	27.016
15	0.106	0.236	0.16	0.168	0.152	−9.748
16	0.076	0.241	0.154	0.172	0.118	−31.092
17	0.116	0.431	0.198	0.179	0.289	60.805
18	0.062	0.129	0.098	0.096	0.094	−1.45
19	0.08	0.298	0.14	0.098	0.08	−19.156
21	0.083	0.29	0.135	0.104	0.141	36.39

## Data Availability

The data used in this study is available in the following repository: https://gitlab.di.unipmn.it/Christopher.Irwin/acceleromerer-data (accessed on 28 August 2023).
